# A case of effective tocilizumab for arthropathy due to dialysis-related amyloidosis

**DOI:** 10.1007/s13730-025-01009-x

**Published:** 2025-06-26

**Authors:** Susumu Tsunoda, Yusuke Yoshimura, Yuki Oba, Daisuke Ikuma, Hiroki Mizuno, Masayuki Yamanouchi, Tatsuya Suwabe, Izuru Kitajima, Kei Kono, Kenichi Ohashi, Yoshifumi Ubara, Naoki Sawa

**Affiliations:** 1 Department of Nephrology, Nephrology Center, Toranomon Hospital, 2-2-2, Toranomon, Minato-ku, Tokyo 105-8470 Japan; 2 Department of Orthopaedic Surgery, Toranomon Hospital, Minato-ku, Tokyo 105-8470 Japan; 3 Department of Diagnostic Pathology, Toranomon Hospital, Minato-ku, Tokyo 105-8470 Japan; 4 Department of Human Pathology, Institute of Science Tokyo, Bunkyo-ku, Tokyo 113-8510 Japan

**Keywords:** Dialysis-associated amyloidosis, Tocilizumab, Joint ultrasound, Seronegative rheumatoid arthritis

## Abstract

A 79-year-old male patient on hemodialysis for 29 years was admitted to the hospital with unexplained fever (C-reactive protein, 16.1 mg/dL), anemia (hemoglobin 7.6 g/dL), and multiple joint swelling that began 7 months earlier. Infection was ruled out, both rheumatoid factor and anti-cyclic citrullinated peptide antibodies were negative. Positron emission tomography–computed tomography showed positive findings in bilateral shoulder joints, palmar region, femoral head area, and cervical spine. Magnetic resonance imaging showed low intensity on T1 and low-normal intensity on T2, especially in the shoulder joint. Musculoskeletal ultrasound revealed hypoechoic material with surrounding fluid collection and Doppler signals indicating blood flow in the shoulder joint, resembling synovitis associated with rheumatoid arthritis. Biopsy of the shoulder joint mass showed positive Congo-red and direct fast scarlet staining, positive β2-microglobulin, and infiltration of CD68-positive macrophages. Given the history of previous carpal tunnel release and cervical destructive spondyloarthropathy, dialysis-related amyloidosis, and amyloid-related arthritis were diagnosed. However, as the case had the feature of rheumatoid arthritis-like synovitis, tocilizumab was initiated, resulting in improved anemia and Clinical Disease Activity Index. We speculate that dialysis-related amyloidosis can present with arthritis involving cytokines, similar to rheumatoid arthritis, and tocilizumab may be effective for the resulting synovitis.

## Introduction

Various bone lesions have been reported to occur in patients with a prolonged history of dialysis: renal osteodystrophy or chronic kidney disease-mineral and bone disorder related to Ca/P metabolism, and dialysis amyloidosis. It is speculated that β2-microglobulin (β2MG) is deposited in the periosteo-cartilage area due to elevated serum levels of β2MG, and β2MG becomes amyloid fibrils after 5–10 years of dialysis, which are modified by cytokines and macrophages and induce inflammation, resulting in osteoarticular destruction. This is called dialysis-related amyloidosis (DRA). DRA is associated with carpal tunnel syndrome (CTS), destructive spondyloarthritis (DSA), spinal canal stenosis, and bone cyst formation in the femoral head [1–5], and can cause acute arthritis [6]. Whether the articular symptoms in dialysis-related amyloidosis are due to mass effect of amyloid deposition, joint destruction, or inflammatory arthritis has received limited attention. In this report, we present a case of polyarthritis resembling rheumatoid arthritis (RA) complicating dialysis-related amyloidosis in a patient undergoing long-term dialysis, which demonstrated a favorable response to tocilizumab.

## Case presentation

A 79-year-old male started hemodialysis 29 years ago due to renal dysfunction caused by chemotherapy for seminoma. The patient was undergoing online hemodiafiltration (HDF) with a pre-dilution volume of 24 L, using a FIX-210E dialyzer. Each dialysis session lasted 4 h, with a blood flow rate of 250 mL/min and a dialysate flow rate of 600 mL/min. The average pre-admission Kt/V was 1.70. Eight years ago, the patient became aware of numbness in the first through third fingers of both hands and was diagnosed with bilateral CTS. Carpal tunnel release was performed surgically. Seven years ago, numbness appeared in bilateral upper limbs. DSA of the 5th and 6th cervical vertebrae were diagnosed and surgery was performed. DRA was confirmed in both resected specimens. Seven months ago, the patient developed a persistent fever of around 38 °C and a C-reactive protein (CRP) of 5–10 mg/dL. In addition, the patient developed anemia resistant to erythropoiesis-stimulating agents and hypoxia-inducible factor (HIF)-proline hydroxylase-containing protein (PH) inhibitors. The patient was admitted to our hospital for a close examination.

**Fig. 1:**
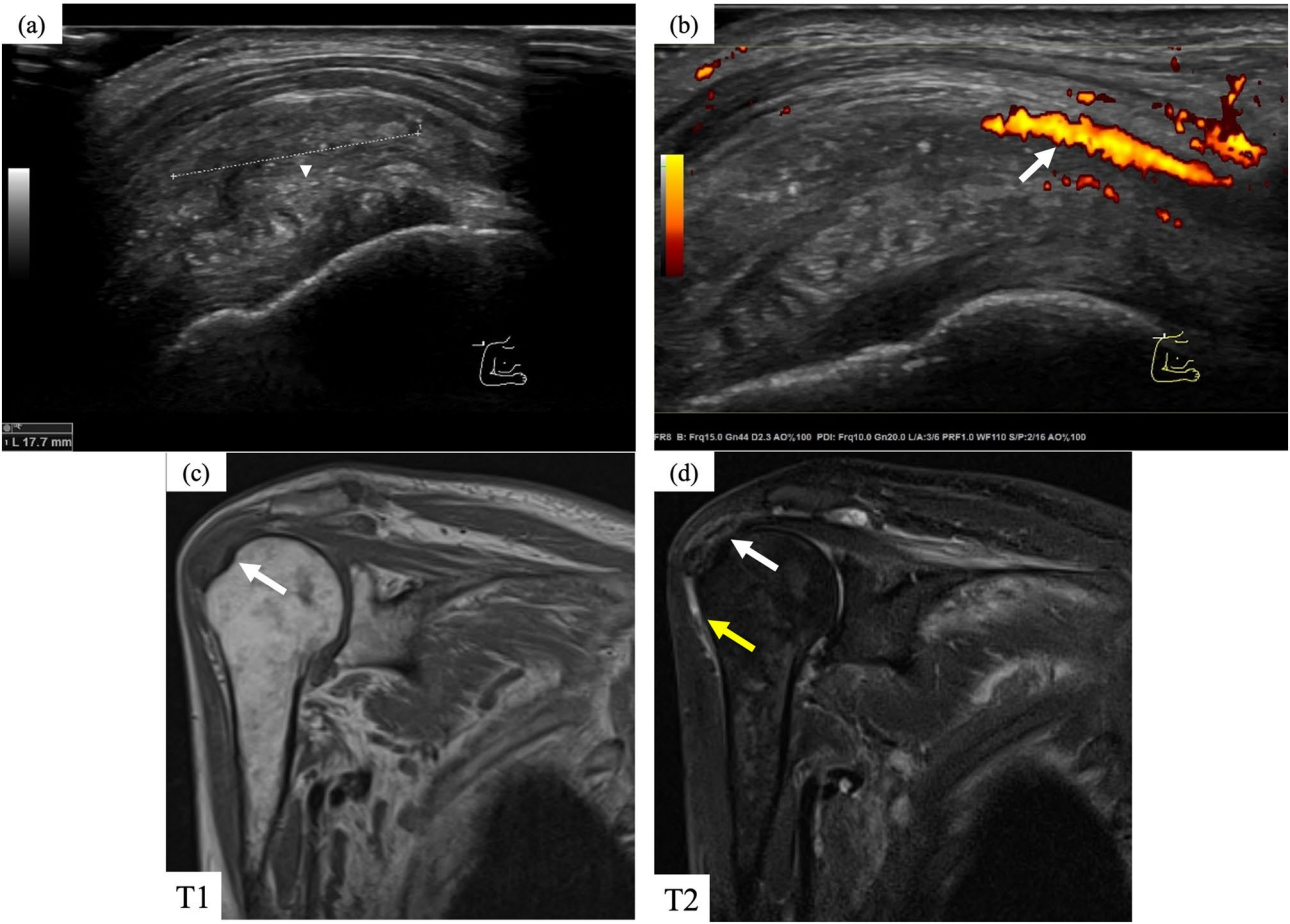
Imaging findings. **a** Ultrasonography of the shoulder joint showed a uniform mosaic echogenic area (white arrowhead) in the subdeltoid smooth muscle. **b** Doppler ultrasonography revealed increased blood flow (white arrow) around the mosaic echo area. **c** MRI showed an area of low intensity (white arrow) on T1-weighted images. **d** MRI showed low to normal intensity (white arrow) on T2-weighted images. In addition, there was an inflammatory image with high intensity (yellow arrow) on T2-weighted images. *MRI* magnetic resonance imaging

**Fig. 2:**
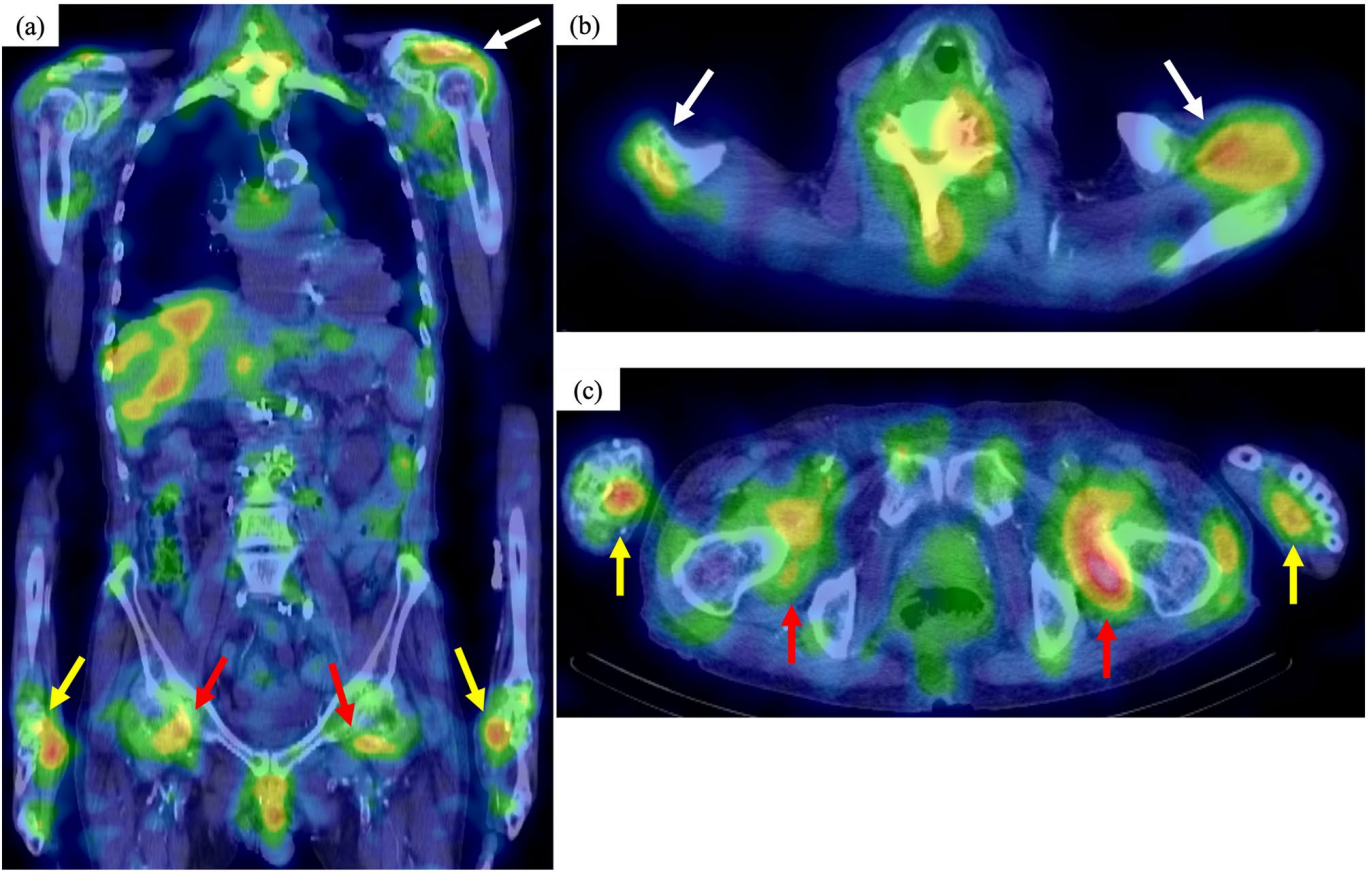
Imaging findings. **a** Coronal view of positron emission tomography-computed tomography image and **b**, **c** transverse views. Intense F-18 fluorodeoxyglucose uptake in masses in the bilateral palmar (yellow arrow), bilateral perifemoral (red arrow), neck, and bilateral shoulder joints (white arrow)

On admission, the patient had pain and swelling of both wrist joints, as well as pain in the hip joint, shoulder joint, and cervical spine. The laboratory findings were as follows: CRP, 16.1 mg/dL; white blood cell, 5000/µL (neutrophil 74.3%); hemoglobin, 7.6 g/dL; platelet, 21.8 × 104/µL; β2-microglobulin, 21.1 mg/dL; IgG, 1435 mg/dL; IgA, 992 mg/dL; IgM, 46.2 mg/dL; IgA-κ M protein, (+); interleukin-6 (CLEIA), 30.6 pg/mL (normal: < 5.8 pg/mL); high sensitivity TNF-α (ELISA), 2.14 pg/mL (normal: 0.75–1.66 pg/mL). The CDAI (clinical disease activity index) was 28. In this case, the treatment was later initiated with tocilizumab; therefore, in accordance with the consensus, disease activity was assessed using the CDAI, which is appropriate for evaluating inflammatory disease activity during IL-6 inhibitor therapy [7]. Shoulder musculoskeletal ultrasonography revealed a uniform, mosaic echogenic pattern within the subdeltoid bursa, suggestive of amyloid deposition and Doppler ultrasonography demonstrated blood flow, indicating significant synovitis (Fig. 1a, b). Magnetic resonance imaging (MRI) revealed a low-intensity area on T1-weighted imaging (Fig. 1c) and low-to-normal intensity on T2-weighted imaging (Fig. 1d) in the shoulder joint, with a small surrounding effusion (Fig. 1d), consistent with previously reported findings of DRA [8, 9]. Positron emission tomography-computed tomography (PET-CT) showed intense F-18 fluorodeoxyglucose uptake in the bilateral palmar, bilateral perifemoral, neck, and bilateral shoulder joints (Fig. 2a–c).

**Fig. 3:**
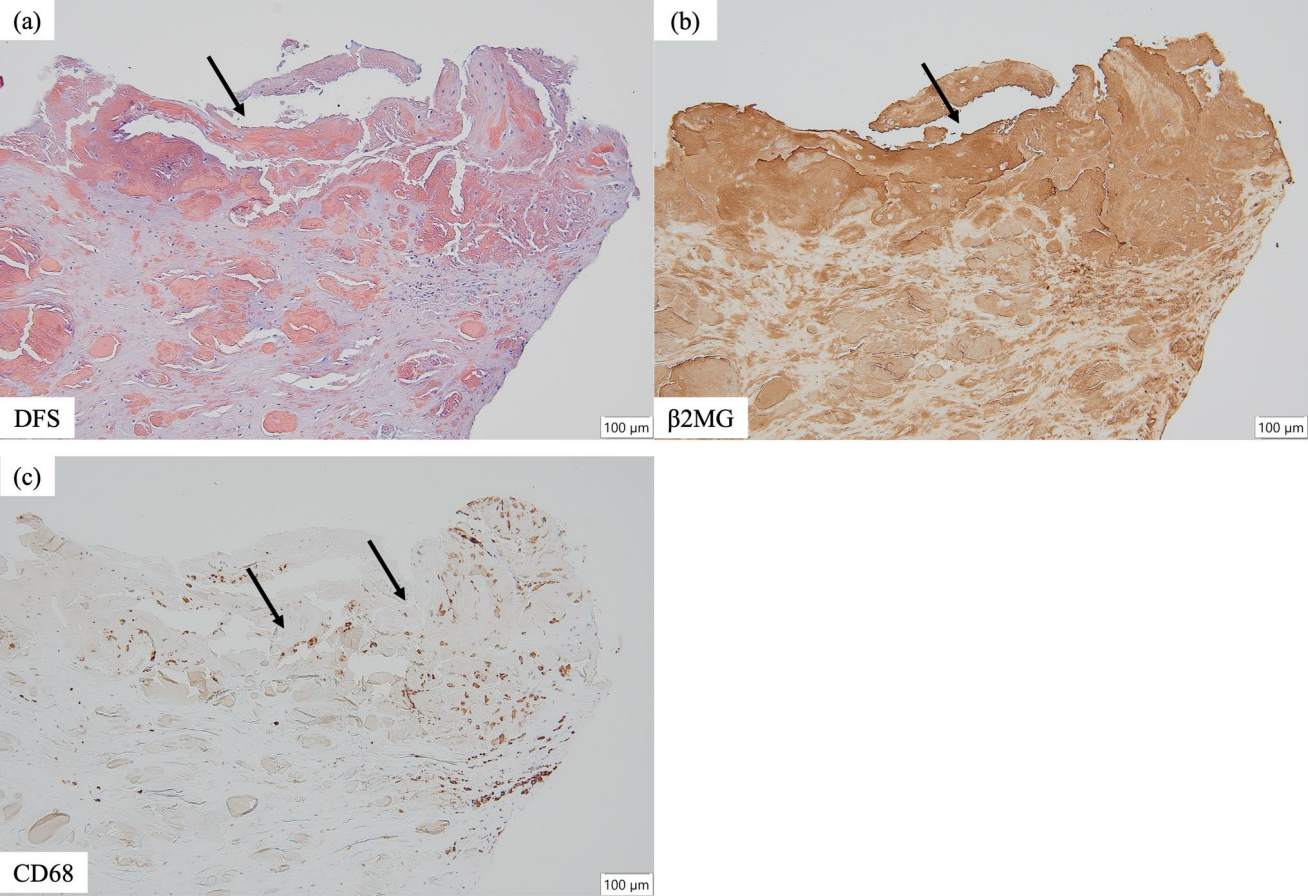
Pathological findings of a mass in the left shoulder joint. **a** Numerous eosinophilic, amorphous structures were observed (black arrow; Congo-red/DFS stain; original magnification, ×100). **b** Similarly, β2-microglobulin-positive eosinophilic amorphous structures were observed (black arrow; β2-microglobulin stain; original magnification, ×100). **c** Numerous CD68-positive macrophages were observed near the Congo-red/DFS and β2-microglobulin-positive structures (black arrow; CD68 stain; original magnification, ×100). *DFS* direct fast scarlet, *CD68* clusters of differentiation 68

**Fig. 4:**
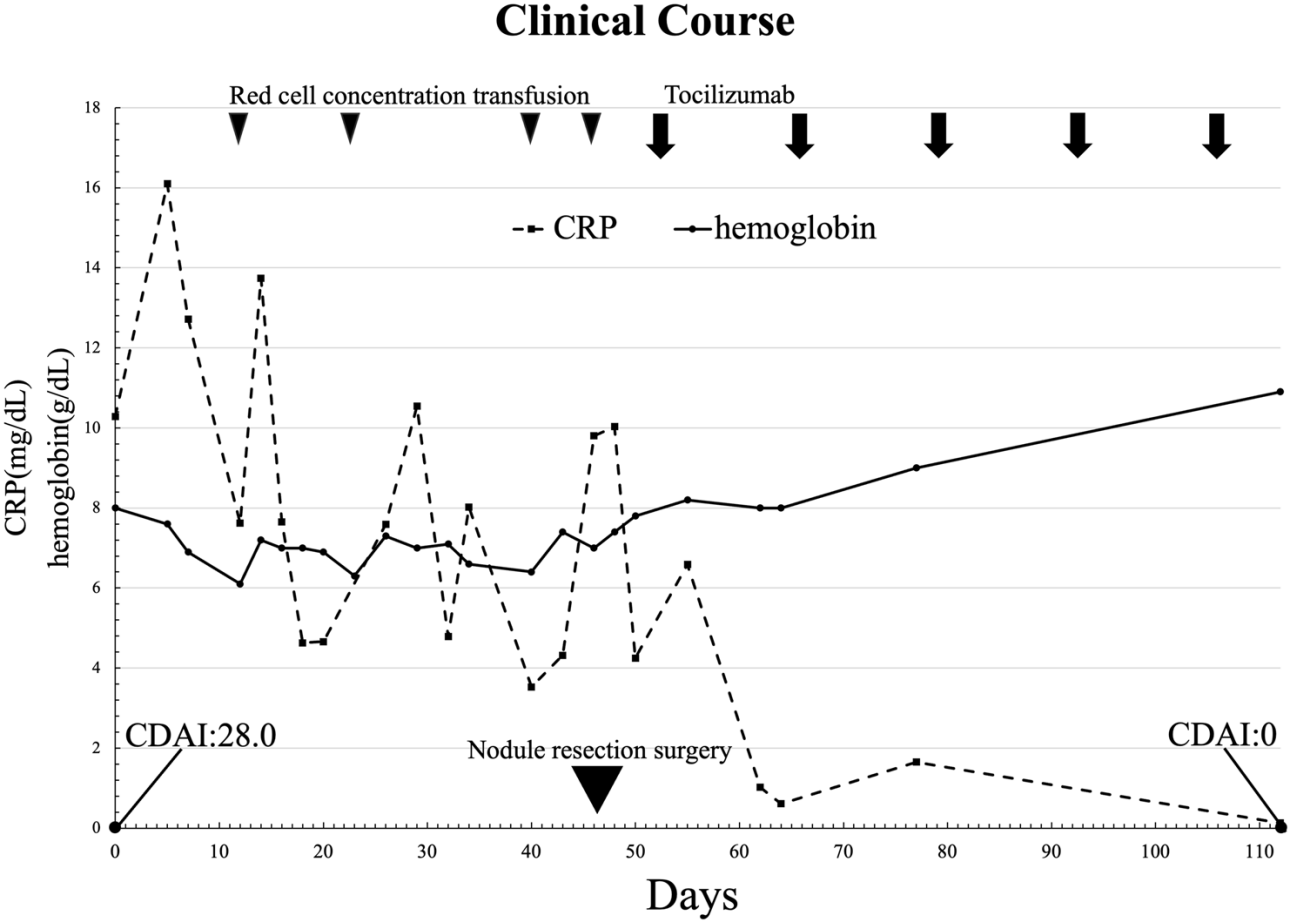
Clinical course. Initially, the patient had a CRP level in the range of 10 mg/dL and anemia that was resistant to erythropoiesis-stimulating agents, requiring transfusions, CDAI was 28.0. The black arrowheads indicate the timing of the transfusion of 2 U of red blood cells. Following the initiation of tocilizumab every 2 weeks, CRP levels became negative, and the patient was able to discontinue transfusions, with hemoglobin levels improving to around 10 g/dL, and CDAI improved to 0. The black arrows indicate the timing of the administration of 162 mg of tocilizumab. One and a half years after the nodule removal, the patient remained free of persistent fever, and hemoglobin levels had further improved to approximately 12 g/dL, and CDAI remained at 0. *CRP* C-reactive protein, *CDAI* clinical disease activity index

The patient also had transfusion-dependent anemia with hemoglobin levels around 8.0 g/dL, which was resistant to erythropoiesis-stimulating agents and HIF-PH inhibitors. Infectious diseases including tuberculosis were ruled out, both rheumatoid factor and anti-cyclic citrullinated peptide antibodies were negative. The blood tests revealed pancytopenia, and the presence of IgA-κ M protein. A bone marrow examination revealed an increase in plasma cells (7%), but no atypia, and the diagnosis of multiple myeloma was excluded. Upper and lower gastrointestinal endoscopy revealed no malignant lesions. Biopsy of the left shoulder joint mass showed Congo red and direct fast scarlet (DFS)-positive amyloid (Fig. 3a), strongly positive for β2-microglobulin staining (Fig. 3b), and negative for κ, λ, transthyretin, and AA amyloid, confirming a diagnosis of β2-microglobulin-associated DRA. Furthermore, CD68-positive macrophages were observed infiltrating the periphery of the amyloid deposits (Fig. 3c). As the case had the feature of rheumatoid arthritis-like synovitis after obtaining informed consent, tocilizumab was initiated for the arthritis, resulting in defervescence, improvement of CDAI 28.0–0, and improvement of anemia to Hb of 10 g/dL, allowing the patient to be discharged without transfusion dependence (Fig. 4). After discharge, we initiated use of a β2-microglobulin adsorption column in addition to ongoing online HDF. Tocilizumab 162 mg every 2 weeks was continued, and after 18 months, no fever or arthritis was observed, and hemoglobin level improved to around 12 g/dL. The CDAI remained at 0, even 18 months after discharge.

## Discussion

We treated a patient with long-term hemodialysis who presented with polyarthritis, elevated CRP, and systemic inflammatory symptoms such as fever, in the context of dialysis-related amyloid deposition characteristic of the shoulder and femoral head. Although arthritis associated with dialysis-related amyloidosis is rarely reported, Ohashi et al. found inflammatory cell infiltrates, primarily CD68-positive macrophages, surrounding DRA deposits, with excessive TNF-α and IL-1β observed histologically within these macrophages [1]. They observed the tissue lesions in detail and inferred the following mechanism. When an amyloid region is formed, inflammatory cells, mainly macrophages, are mobilized as a foreign body response. These cells secrete inflammatory cytokines such as TNF-α and IL-1β, which not only damage intervertebral discs and cartilage in the vicinity of amyloid deposition, but also cause bone destruction through osteoclastic activity of macrophages, resulting in joint and bone lesions [1]. These mechanisms of joint and bone destruction are similar to those in RA. However, the factors that induce cytokine hypersecretion in RA joints are not fully understood.

While MRI has been increasingly used to detect DRA, Cheon et al. demonstrated that PET-CT can also be valuable, identifying hypermetabolic activity and osteolysis in the shoulder, wrist, and lumbar region in a hemodialysis patient with DRA, suggesting its utility in diagnosing DRA-related arthritis [9]. Given the established role of PET-CT in identifying inflammatory sites in RA, it may serve as a comparable indicator of arthritis activity in both DRA and RA [10–12]. In our case, PET-CT and histology confirmed both inflammation at the site of DRA and macrophage infiltration, highlighting the clinical and histological similarities between DRA-associated arthritis and RA with systemic inflammation.

Symptoms consistent with DRA-associated arthritis improved following tocilizumab administration. Regarding the mechanism of inflammation in amyloidosis, pro-inflammatory cytokines such as IL-1β, IL-6, and TNF-α are associated with the formation of amyloid plaques, and these cytokines are proposed to trigger inflammatory responses and promote tissue damage [11]. In our case, IL-6 and TNF-α were elevated as previous reports describe [13, 14]. Recent studies have demonstrated an upregulation of gene expression and activation of transcription factors within the IL-6/STAT3 pathway in anti-citrullinated peptide antibody (ACPA)-negative RA patients, in contrast to those who are ACPA-positive [12, 15, 16]. Therefore, tocilizumab, an anti-IL-6 receptor antibody, may be a beneficial option for managing inflammation in dialysis-related amyloidosis. To our knowledge, this is the first report of tocilizumab for DRA-associated arthritis. Given tocilizumab’s documented efficacy in seronegative RA complicated by AA amyloidosis, reducing serum amyloid A and improving clinical symptoms [17–19], and its successful use in familial Mediterranean fever-associated amyloidosis, we propose that tocilizumab may also be effective for arthritis associated with DRA and systemic inflammation [17, 20].

This case presentation has a notable limitation: we were unable to provide objective imaging evidence—such as follow-up musculoskeletal ultrasound or PET-CT—to confirm the resolution of synovitis after treatment. This is because tocilizumab therapy was continued at a different institution following the patient’s discharge and post-treatment imaging studies were not obtained. However, the patient was followed clinically and physical examinations were consistently documented. In this case, disease activity was assessed using CDAI, which is considered appropriate for evaluating inflammatory disease activity during IL-6 inhibitor therapy. A consensus statement on IL-6 receptor inhibition recommends the use of composite indices that exclude acute-phase reactants—such as the CDAI—for assessing disease activity in both rheumatoid arthritis and other inflammatory conditions [7]. Notably, the patient’s CDAI improved from 28 to 0 and transfusion-dependent anemia related to chronic inflammation resolved. Although the absence of imaging precludes definitive confirmation of arthritis remission, the improvement in symptoms suggests that tocilizumab may have been effective in alleviating clinical manifestations of arthritis.

In summary, we speculate that dialysis-related amyloidosis can present with arthritis involving cytokines, similar to RA, and tocilizumab may be effective for the resulting synovitis.

## Data Availability

The datasets used and/or analyzed in the current study are available from the corresponding author upon reasonable request.
